# Elevating the University Teaching Qualification: From Ticking the Box to Actual Impact on Teaching

**DOI:** 10.5334/pme.2359

**Published:** 2026-06-05

**Authors:** Felicitas Biwer, Lianne M. Loosveld, Boukje Compen

**Affiliations:** 1 Department of Educational Development and Research, School of Health Professions Education (SHE), Maastricht University, Maastricht, the Netherlands

## Abstract

**Background & Need for Innovation:**

At the medical faculty of a Dutch university, about 85 teachers annually participate in the University Teaching Qualification (UTQ) trajectory. Although the trajectory had long remained unchanged, evaluations showed that many joined primarily to obtain certification, with limited intrinsic motivation. The assessment portfolio was experienced as burdensome and insufficiently reflective.

**Goal of Innovation:**

We aimed to redesign the UTQ trajectory to foster not only qualification but also sustainable professional growth, better aligned with teachers’ daily educational practice.

**Steps taken for Development and Implementation of Innovation:**

The trajectory was revised iteratively. The portfolio gained a more integrated role as participants were encouraged to develop it continuously during and between sessions, supported by formative feedback. This differed from the previous approach, where participants often did not engage in reflection on their teaching until after the final session. Furthermore, coherence across sessions was strengthened, and links to participants’ own practices were enhanced through competency-based assignments and a personal project involving the (re)design of their own educational activity.

**Outcomes of Innovation:**

Through questionnaires and interviews, participants (N = 83) reported gained knowledge and confidence and felt the trajectory was practically relevant. The integrated portfolio enhanced reflection and growth, while peer learning, microteaching, and frequent feedback supported continuous learning and innovation.

**Critical Reflection on Process:**

The iterative redesign illustrated that innovation in faculty development can emerge through incremental, feedback-driven change. Evaluations from participants and facilitators guided adjustments to sequence, assignments, and support. Close collaboration between program designers and facilitators enabled rapid improvements.

## Background & Need for Innovation

Mission statements of universities around the world reflect the aim of providing quality education [1]. Nevertheless, it used to be common practice for university staff to be allowed to teach without having obtained a teaching qualification [2]. Institutions increasingly started offering faculty development, also referred to as teacher professional development, staff development or teacher training [3], to support staff in developing their didactic competencies. However, the voluntary nature of such initiatives meant that effective teaching could not be guaranteed [4]. Norway, Sweden and the United Kingdom were among the first countries to introduce compulsory faculty development for academic staff involved in teaching [5,6,7]. The Netherlands followed in 2008, when all universities – encouraged by student unions and the Ministry of Education, Culture and Science – agreed to introduce the University Teaching Qualification (‘UTQ’) [8], serving as proof of one’s didactic competencies.

Each Dutch university offers a trajectory to support staff in obtaining this qualification. To enable mutual recognition of the UTQ within the Netherlands, the trajectories in all universities capture the same competences: designing education, delivering and evaluating education, and supervising and assessing students [9]. Furthermore, assessment of the UTQ is based on participants’ portfolios, demonstrating the progression of their teaching competencies over time [2,10]. Nevertheless, universities have a certain degree of autonomy in the exact format and content of the UTQ and portfolio [10].

At Maastricht University, Faculty of Health, Medicine and Life Sciences approximately 85 staff members participate in the UTQ trajectory on a yearly basis. Besides some minor changes and a shift to a flipped classroom approach during the COVID-19 pandemic, the set-up of the UTQ had largely remained the same since its start. The trajectory started with an intake to assess eligibility to the trajectory (based on teaching experience and size of teaching appointment). As described earlier in Schreurs et al. [11], participants then attended five training days, spread over a five-month period. A different UTQ competence took center stage during each training day. Aligned with the university’s focus on problem-based learning (PBL) [12], the sessions took place in groups of maximum 12 participants, facilitated by a faculty developer. The sessions were highly interactive and the composition of groups remained the same throughout the trajectory to encourage community building [11,13]. In the portfolio, participants described and reflected on three teaching cases per competency and related these to educational literature. Participants’ portfolios were assessed by a faculty developer and a staff member with an educational leadership position. Additional questions about the portfolio were asked during an interview.

The decision to start revising the UTQ in 2023 was due to several reasons. The UTQ used to primarily target “teachers and […] faculty members who want to improve their educational skills and expertise” [11, p. 810]. However, the fact that it is a prerequisite for being accepted for or promoted to certain academic positions [8,10], seemed to have increased the number of participants attending the trajectory for the sake of ‘ticking the box’. Another reason stemmed from formal (e.g., evaluation surveys) and informal (e.g., group discussions) participant evaluations of the trajectory. These increasingly started revealing that although participants enjoyed the sessions and felt these were valuable to improving their teaching, the composition of the portfolio was perceived as a demotivating and time-consuming task. This often resulted in postponing portfolio writing until after the final group session. As faculty developers, we noticed that this retroactive approach impaired participants’ learning and made it challenging to compose a coherent portfolio that clearly demonstrated how participants’ educational competencies had improved due to the UTQ.

## Goal of Innovation

One goal of our innovation related to the experience that an increasing number of participants completed the UTQ because they ‘had to’ rather than because they ‘wanted to’. Therefore, we aimed to enhance the intrinsic value of the UTQ trajectory. In particular, our goal was to create a learning experience that was relevant to staff members’ own educational practices and that met their personal development needs. In light of participants’ evaluations and faculty developers’ impressions of the portfolios, the second goal of the innovation was to increase meaningful engagement and competence development of participants by adjusting the requirements and format of the portfolio.

Combining these two goals, we aimed for a UTQ trajectory that could result in win-win situations: if the time and effort participants invest in the portfolio could lead to a concrete outcome directly relating to their own educational practices, we assumed that the trajectory would not only lead to qualification but would also be perceived as more meaningful. The underlying theoretical principles on which we based this assumption are discussed in the next section.

## Steps taken for Development and Implementation of Innovation

The UTQ trajectory was revised in an iterative process following the ADDIE instructional design model, including the phases of Analysis, Design, Development, Implementation and Evaluation. Although the origin of ADDIE is unclear, it is widely used to (re)design education in a structured and iterative way [14], which suited our intentions. The revision process was conducted by a team of four faculty developers who had been facilitating UTQ groups for years.

Based on the analysis and needs for change identified, we determined what participants should learn in the revised UTQ, which types of activities would best support this, and how their learning would be evaluated. In other words, we revised the intended learning outcomes (ILOs), teaching and learning activities (TLAs) and assessment of the trajectory. In line with the concept of constructive alignment [15] – regarded as a key principle of high-quality educational design – we ensured that these elements were coherent. For an overview of the ILOs, see Appendix 1. In the next paragraphs, we elaborate on the TLAs and assessment in the revised UTQ.

The core of the revised UTQ remained to evolve around five training days spread over half a year. This structure was kept to adhere to organizational regulations but is also grounded in literature encouraging longitudinal involvement in faculty development [16]. Each day now addressed at least two of the competences that were set at institutional level (educational design, assessment, teaching delivery, professional conduct). The design of competence-specific TLAs was informed by the four learning principles underlying the university’s PBL philosophy [12]. In particular, it was ensured that the TLAs included elements of collaborative learning (e.g., via peer discussions and microteaching sessions), constructive learning (e.g., via reflective homework assignments applying theory and connecting to prior knowledge), contextual learning (e.g., via authentic assignments and application to own educational practice), and self-directed learning (e.g., letting participants guide their own learning by their own learning goals and regular monitoring and reflecting).

The assessment was changed from being purely summative to including formative aspects too. These aspects were aimed at providing participants with insight into their current competencies and how these could be further developed [17] but also prevented that portfolio writing would be postponed until after the final session. Participants worked on four competence-specific assignments between the training days:(i) making an ADDIE analysis for one of their educational roles, (ii) analyzing different assessment formats in their course, (iii) applying an instructional design model to their educational practices, and (iv) reflecting on their team roles in educational teams. Questions and reflections on these competence-specific assignments were discussed in the groups. The combined assignments form the first part of the portfolio.

Additionally, throughout the trajectory and in line with the principle of contextual or authentic learning [12], participants worked on a project aiming to improve and innovate their own educational practices using what they learned during the trajectory. Each training day, participants discussed their projects and received intermediate feedback from peers and staff. A description of and reflection on the educational project formed the second part of the portfolio. By actively encouraging participants to continuously work and reflect on the project and portfolio during the trajectory, we aimed to prevent artificial reflection and enable shorter feedback loops. This approach could additionally support participants in their roles as educational innovators. The complete portfolio and project were assessed summatively by two assessors and completed with a final presentation and interview.

In January 2024, the first revised UTQ trajectory was piloted with a subgroup (n = 22) of the total participant cohort (N = 40). It was delivered in two groups by two faculty developers that had been involved in the redesign. This pilot was carefully evaluated by participants and the team of faculty developers to inform further revisions. Participants were asked to share their experiences during the training days and through a post-UTQ questionnaire. Throughout the trajectory, faculty developers briefly evaluated each individual session and determined whether any changes were required for the next session(s). Once the faculty developers had assessed the first portfolios and presentations, they held various dedicated meetings to discuss revisions they considered valuable for the next trajectory iteration.

**Figure 1:**
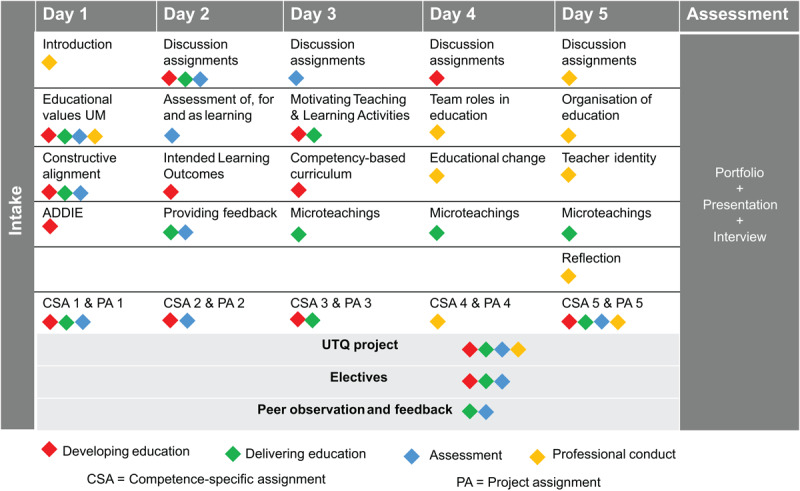
Overview of Elements of UTQ showing the different competences covered.

Revisions made in the second iteration included peer observations and feedback on teaching moments, as well as adding nine electives that participants could choose from to further build their teaching competences and tailor the UTQ content to individual needs. Electives were either offered as self-paced online modules or as group workshops. Additionally, participants received personal feedback from their UTQ teacher on their competence-specific assignments to improve the learning effect and formative aspect of these assignments. The second redesigned trajectory was fully implemented for the full cohort in May 2024 (n = 39) and January 2025 (n = 44). See Figure 1 for an overview of the elements of the final design of the UTQ.

## Evaluation of Innovation

To evaluate the revised version of the UTQ, we used an adapted version of the questionnaire by Vreekamp et al. [18]. Additionally, we invited participants to share their experiences in one-on-one interviews during a 3-month window after the final UTQ session. The questionnaire consisted of five-point Likert scale items assessing different aspects of the trajectory and its impact: knowledge, teaching attitude, perceived behavior change, as well as practicality and relevance of the trajectory. Participants were asked to complete the questionnaire during the final hour of the fifth UTQ session, resulting in a response rate of 75%. For an overview of the mean, SD, and reliability scores for each scale, see Appendix 2.

Overall, participants reported to have gained knowledge by participating in the UTQ course (*M* = 4.17, *SD* = 0.57) and feeling willing and confident to improve their educational practices (*M* = 4.04, *SD* = 0.64). Changes in teaching behavior were less evident at time of the questionnaire (*M* = 3.53, *SD* = 0.70). The course was evaluated well on practicality aspects (*M* = 3.99, *SD* = 0.63) and perceived as relevant (*M* = 4.01, *SD* = 0.77).

The interviews gave more insight into the working ingredients of the UTQ course and suggestions for improvement. One aspect perceived as highly valuable was the connection between educational theory and participants’ own educational practices. Participants described to have gained a better understanding of ‘*how education works’*. The UTQ offered them ‘*a toolbox’* and ‘*the language to apply educational theory to practice’*. Whereas before the UTQ, participants engaged in education by ‘*just doing something*’, what they learned enabled them to think about and design education and assessment more meaningful. As one participant mentioned: “*I do think that going through the whole UTQ process makes you more confident in your role. […] I’ve become more aware of how I do things*.”

One aspect that contributed to participants’ learning was the contextual learning principle. The fact that participants could work on their own educational project and could apply the competence-specific assignments to their context created more meaningful and sustained learning experiences. As one participant described: “*The combination of the theories you get in the sessions, plus then actually putting them into practice really makes it stick. Because I notice that I still remember which theories were discussed and what they mean*.”

Furthermore, participants appreciated the focus on their personal development as teachers through individual and peer feedback. The portfolio was perceived as critical reflection and reference tool for later use, in which participants collected evidence and feedback, and could look back at their growth throughout the trajectory. The fact that the presentation was scheduled at a later point in time enabled participants further to reflect on their learning take aways: “*It’s really only when you start making the final presentation that you truly stop and think: okay, what do I want to take with me? What have I learned? And which parts of my reflection are really important to me? It’s not so much: ‘this makes the portfolio complete,’ but more: ‘what do I actually want to convey?’ Yes — and what have I really learned from it. So I think that was very valuable.”*

Another aspect highly valued was the collaboration between participants, which we facilitated through peer feedback and discussions as well as microteaching sessions in which participants provided a snapshot of their teaching and received feedback. Groups were diverse regarding teaching roles, domain expertise and teaching experience which contributed to diverse learning experiences: *“Just being part of a group: being together*, *giving each other tips and ideas, and sharing thoughts… You really learn a lot from that”*. Important here was the creation of a safe atmosphere and that the same group followed the UTQ trajectory together. As one participant mentioned: “*It also meant that effort was put into creating a good atmosphere, a safe environment where everyone could share. That really encouraged us to listen to others as well. And even though everyone comes from very different disciplines, in the UTQ you still work together… you can still understand each other.”*

Despite these positive experiences, some participants had expected to improve their teaching delivery competence more. They experienced the UTQ focusing strongly on educational design and assessment, and had expected *“When you have a UTQ, that would mean you’re officially qualified to teach. So then you can teach better […]. Well, I actually haven’t experienced that I really learned that.”*

The individual projects of participants showed diversity in scope and embedding in educational practice, depending on participants’ educational roles and responsibilities. Nevertheless, projects overall were remarkably creative and innovative. In Appendix 3, we share two examples of participants’ projects to showcase their nature and scope.

## Critical Reflection on your Process

Guided by our aim to create a win-win situation by improving alignment between the portfolio and competences, as well as making the trajectory more adaptive and relevant for teachers, we decided to give the portfolio a central role. After the first iteration of working with the design, we made minor adjustments to the alignment between the different days, based on feedback from both participants and faculty developers, alongside other refinements such as continuous feedback, peer observations and the introduction of electives. This stepwise and iterative approach following the ADDIE model allowed us to quickly iron out minor kinks without needing to overhaul the entire trajectory. A drawback of this approach was that it required ongoing adjustments to an existing trajectory; however, for us, this was outweighed by the flexibility it offered in meeting participants’ needs. It does, in our eyes, also require that the faculty developers involved in (re)designing the trajectory are closely engaged in its delivery, and vice versa.

Another meaningful insight for us was that the introduction of the individual projects enabled participants to become involved and acquainted with educational design, assessment and delivery and apply it in their own way. This proved to be a valuable example of educational innovation coming bottom-up from more early-career teachers and not top-down from the program management, encouraging teachers to contribute to educational design and not only to deliver it. Several UTQ participants have already been able to put their personal project into practice, but we are also aware of the fact that this will not be (immediately) possible for everyone.

Through our evaluations, we further learned that, referring to the constructive alignment of our trajectory, expectation management needs improvement. Some participants had expected that the UTQ would focus on further improving their teaching delivery, while educational design, assessment and professional conduct also received a lot of attention. We made this choice as specific teaching delivery competences are covered in role trainings that should have been attended prior to participating in the UTQ. Nevertheless, this could be communicated more clearly.

Additional steps to further advance the UTQ include expanding the range of electives. These could be based on participants’ requests but also address key developments affecting education, such as sustainability, social safety, the implications of AI for educational design and assessment, and diversity, equity, inclusion. Furthermore, the more integrated nature of the portfolio could provide additional future opportunities for personalised learning via so-called “Teacher Milestones” – comparable to “Clinician Educator Milestones” [19].

Rather than aiming for a perfect design from the outset, our approach highlights the value of learning through doing, adjusting, and co-creating with participants and faculty developers. By embracing a flexible and participatory design process, guided by user feedback, we hope to have innovated our UTQ trajectory.

## Use of Artificial Intelligence

GPT-5 (OpenAI, September 2025) was used to provide additional English spelling and grammar support to the Dutch authors of this manuscript. The image in Appendix 3b was translated from Dutch to English with help of Google Lens and Google Translate.

## Additional File

The additional file for this article can be found as follows:

10.5334/pme.2359.s1 Appendices. Appendix 1 to 3.

## References

[1] Breznik K, Law KMY. What do mission statements reveal about the values of top universities in the world? Int J Organ Anal. 2019;27(5):1362–1375. DOI: 10.1108/IJOA-08-2018-1522

[2] Molenaar WM, Zanting A. Experiences with the implementation of a national teaching qualification in university medical centres and veterinary medicine in the Netherlands. Perspect Med Educ. 2015;4:43–46. DOI: 10.1007/s40037-015-0159-y 25609171 PMC4348223

[3] Van Schalkwyk S, Amaral E, Anakin M, Chen R, Dolmans D, Findyartini A, et al. Disentangling faculty development: A scoping review towards a rich description of the concept and its practice. Med Teach. 2024;47(8):1304–1325. DOI: 10.1080/0142159X.2024.2429612 39674914

[4] van Bruggen L, ten Cate O, Chen HC. Developing a novel 4-C framework to enhance participation in faculty development. Teach Learn Med. 2020;32(4):371–379. DOI: 10.1080/10401334.2020.1742124 32251617

[5] Lycke KH. Faculty development and issues in a Norwegian perspective. Int J Acad Dev. 1999;4(2):124–133. DOI: 10.1080/1360144990040207

[6] Lindberg-Sand Å, Sonesson A. Compulsory higher education teacher raining in Sweden: Development of a national standards framework based on the scholarship of teaching and learning. Tert Educ Manag. 2008;14(2):123–139. DOI: 10.1080/13583880802053051

[7] Trowler P, Bamber R. Compulsory higher education teacher training: Joined-up policies, institutional architectures and enhancement cultures. Int J Acad Dev. 2005;10(2):79–93. DOI: 10.1080/13601440500281708

[8] de Jong RAH, Mulder JA, Deneer PMH, van Keulen J. Poldering a teaching qualification system in Higher Education in the Netherlands: a typical Dutch phenomenon. REDU: Revista de Docencia Universitaria. 2013;11:23–40. DOI: 10.4995/redu.2013.5517

[9] Association of Universities in the Netherlands. Professionalisation of university lecturers: The UTQ and beyond; 2018.

[10] Brouwer N, Joling E, Kaper W. Effect of a person-centred, tailor-made, teaching practice-oriented training programme on continuous professional development of STEM lecturers. Teach Teach Educ. 2022;119. DOI: 10.1016/j.tate.2022.103848

[11] Schreurs M-L, Huveneers W, Dolmans D. Communities of teaching practice in the workplace: Evaluation of a faculty development programme. Med Teach. 2015;38(8):808–814. DOI: 10.3109/0142159X.2015.1112892 26610150

[12] Dolmans DHJM. How theory and design based research can mature PBL practice and research. Adv Health Sci Educ. 2019;24:879–891. DOI: 10.1007/s10459-019-09940-2 PMC6908548 31720879

[13] Steinert Y. Faculty development: From workshops to communities of practice. Med Teach. 2010;32: 425–428. DOI: 10.3109/01421591003677897 20423263

[14] Molenda M. In search of the elusive ADDIE model. Perf. Improv. 2015;42(5):34–36. DOI: 10.1002/pfi.4930420508

[15] Biggs J. Enhancing teaching through constructive alignment. High Educ. 1996;32:347–364. DOI: 10.1007/BF00138871

[16] Dijk SW, Findyartini A, Cantillon P, Cilliers F, Caramori U, O’Sullivan P, et al. Developing a programmatic approach to faculty development and scholarship using the ASPIRE criteria: AMEE Guide No. 165. Med Teach. 2024;46(6):732–745. DOI: 10.1080/0142159X.2023.2259062 37783204

[17] Black P, Wiliam D. Developing the theory of formative assessment. Educ Assess Eval Account. 2009;21(5):5–31. DOI: 10.1007/s11092-008-9068-5

[18] Vreekamp M, Runhaar P, Gulikers J, den Brok P. Collaboration activities in pedagogical development programmes in higher education: what do teachers learn from this? High Educ Res Dev. 2025;44(5):1225–1241. DOI: 10.1080/07294360.2025.2472840

[19] Accreditation Council for Graduate Medical Education, Accreditation Council for Continuing Medical Education, Association of American Medical Colleges, Medicine. Clinician educator milestones; 2022. Available from: https://www.acgme.org/globalassets/pdfs/milestones/standalone/2022/clinicianeducatormilestones. 10.1093/acamed/wvaf044 41685756

