# Supplementary material for: Elevating the University Teaching Qualification: From Ticking the Box to Actual Impact on Teaching
Source: Perspect Med Educ. 2026 Jun 5;15(1):475–81. doi: 10.5334/pme.2359 (PMC13239334; doi:10.5334/pme.2359)
Supplement: Appendices. — Appendix 1 to 3. [file pme-15-1-2359-s1.pdf]

Appendix 1. Intended Learning Outcomes

Competence	ILO
Developing education<br>UTQ-participant can:	1. Apply the educational principles of active, student-centred learning in the development of education<br>2. Explain the position of their part of the curriculum in relation to the structure and the intended learning outcomes (ILOs) of either the entire curriculum or the relevant part of it<br>3. Justify the educational design choices made for different parts of the curriculum, such as a course, workshop, training session(s) or lecture<br>4. Identify characteristics of their target group, such as prior knowledge, and design education based on these characteristics<br>5. Clearly express and communicate the ILOs to students<br>6. Align the ILOs, teaching and learning activities (TLAs) and assessment methods using the principles of constructive alignment<br>7. Improve education based on student evaluations, teaching experience and recent educational insights
Delivering education<br>UTQ-participant can:	8. Apply the educational principles of active, student-centred learning in teaching<br>9. Encourage students to embrace the educational principles of active, student-centred learning<br>10. Supervise students individually and in groups and provide constructive feedback<br>11. Analyse strengths and weaknesses of own teaching performance and to adjust activities accordingly
Assessment<br>UTQ-participant is:	12. Familiar with the assessment policy, the education and examination regulations (EER), and the rules and regulations (R&R) of the faculty<br>13. Able to choose and implement an appropriate assessment method based on the principles of constructive alignment<br>14. Familiar with the difference between assessment of, for and as learning and able to apply these principles effectively<br>15. Able to give students constructive feedback based on formulated assessment criteria<br>16. Familiar with the assessment cycle and able to justify the choices made in each step
Professional conduct<br>UTQ-participant is:	17. Aware of own educational and teaching vision and how it aligns with the educational vision of UM, the faculty and the relevant degree programs<br>18. Acquainted with how teaching activities are evaluated, and able to interpret the results to improve education<br>19. Able to work together in an educational team to develop and coordinate teaching activities<br>20. Able to give constructive feedback to colleagues on their education and teaching<br>21. Able to reflect on developed education and the delivered teaching based on requested feedback in order to improve<br>22. Able to set goals for further professional development as a teacher

Appendix 2. Results of the evaluation survey.

As a result of participating in UTQ activities…	M	SD	N
I have more knowledge about teaching models and theories	4.53	0.60	60
I have more knowledge about how to design good education	4.37	0.78	60
I have more knowledge about how to deliver education	3.82	1.03	60
I have more knowledge about assessment	4.12	0.78	60
I have more theoretical understanding of what good assessment is	4.18	0.68	60
My role as a teacher is clearer to me	3.98	0.83	60
Knowledge scale	***4.17***	***0.57***	***60***
My own vision on teaching and learning is clearer for me	3.83	0.94	60
I am more willing to innovate my teaching	4.23	0.83	60
I am more willing to use other educational tools in education	4.05	0.96	60
I am more willing to continuously try to improve my education	4.17	0.87	60
I feel more confident as a teacher	3.67	0.95	60
I feel able to apply what I learned during the UTQ course in my educational roles	4.25	0.68	60
I feel confident about applying what I learned during the UTQ course in my educational roles	4.10	0.71	60
Attitude scale	***4.04***	***0.64***	***60***
I prepare my teaching activities differently	3.54	0.93	59
I (re)design my teaching materials	3.75	0.86	59
I (re)design the setup of my course	3.47	0.95	59
I (re)design the assessment of my course	3.36	1.01	59
I behave differently in teaching situations	3.53	0.88	59
I act more in line with my vision on teaching and learning	3.59	0.85	59
I act differently in engaging with my students	3.47	0.88	59
Behaviour scale	***3.53***	***0.70***	***59***
The content of the UTQ course was easy for me to follow	4.08	1.08	60
The content of the UTQ aligned well with my prior knowledge	3.70	1.03	60
I had sufficient time to ask questions during the UTQ course	4.72	0.64	60
I had sufficient time to perform the assignments during the UTQ course	3.98	1.08	60
The length of the sessions was adequate	3.75	1.18	59
The scope of the portfolio assignments was adequate	3.57	1.00	60
The extent to which my teacher competencies developed justifies my time investment in the UTQ	3.47	1.24	60
The group discussions contributed to my learning	4.23	0.91	60
The collaboration with other participants has been productive for my learning	4.30	0.81	60
I enjoyed participating in the UTQ course	4.13	0.97	60
Practicality scale	***3.99***	***0.63***	***60***
I have learned a lot during the UTQ course	3.95	1.05	60
The content of the UTQ sessions was relevant to me	3.98	0.97	60
The content of the UTQ course is applicable to my educational roles	4.05	0.79	60
What I learned during the UTQ course will help me to fulfil my educational	4.13	0.89	60

roles better			
The assignments I performed in the UTQ course are relevant to my educational roles	3.92	0.91	60
Relevance scale	***4.01***	***0.77***	***60***

**Why did you enrol in the UTQ program? (multiple answers possible)**	freq	%
Because the UTQ is required for the teaching I do or want to do in the future	38	63
Because the UTQ is required by my department.	15	25
Because the UTQ is crucial for my career.	35	58
Because I want to improve my teaching.	42	70
Other	4	7

Appendix 3a. Project

Virtual Reality and 360 Degree Video in Medical Education

Context and educational problem
The participant chose to focus their UTQ project on a module called ‘abdomen’, offered in the third year of the Bachelor in Medicine. The identified educational problem in this module was that clinical reasoning was primarily taught through discussion of written cases, rather than through authentic exposure to real patients and clinical settings.

Proposed innovation

Development of VR/360° videos depicting authentic clinical scenarios (outpatient clinic, emergency department, ultrasound, surgery) focusing on (complicated) gallstone disease. In particular, students would engage in a two-hour workshop in which they would watch the videos, followed by a group discussion and clinical reasoning guided by a tutor, reflection, and an expert session. Integration of the innovation was expected to enhance constructive alignment within the module, as well as to foster student motivation as it addresses various components of the Self-Determination Theory and the Attention, Relevance, Confidence and Satisfaction model.

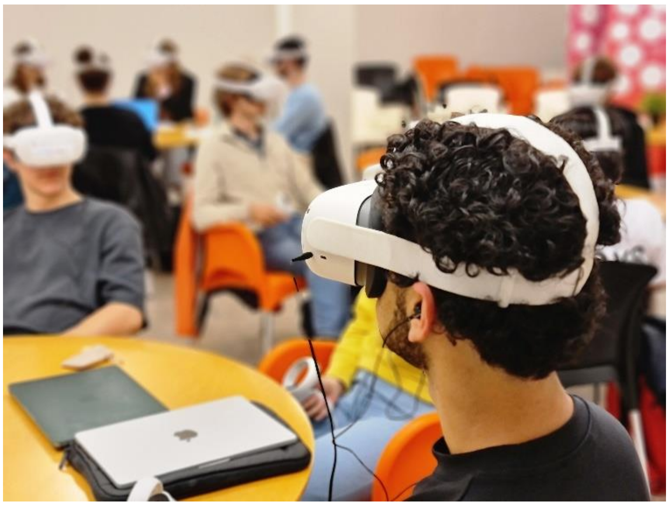

Implementation, evaluation and future vision
The pilot was launched during the 2024–2025 academic year and evaluated by both students and tutors. In particular, questionnaire and focus groups were held to evaluate perceived effectiveness, logistics, and student motivation. Depending on the evaluations, the VR-based teaching could be expanded to other clinical topics (dyspnoea, chest pain, resuscitation, trauma) and use in other programs (Health Sciences, Biomedical Engineering). Currently, it will be implemented in the Bachelor and Master Programme of Medicine.

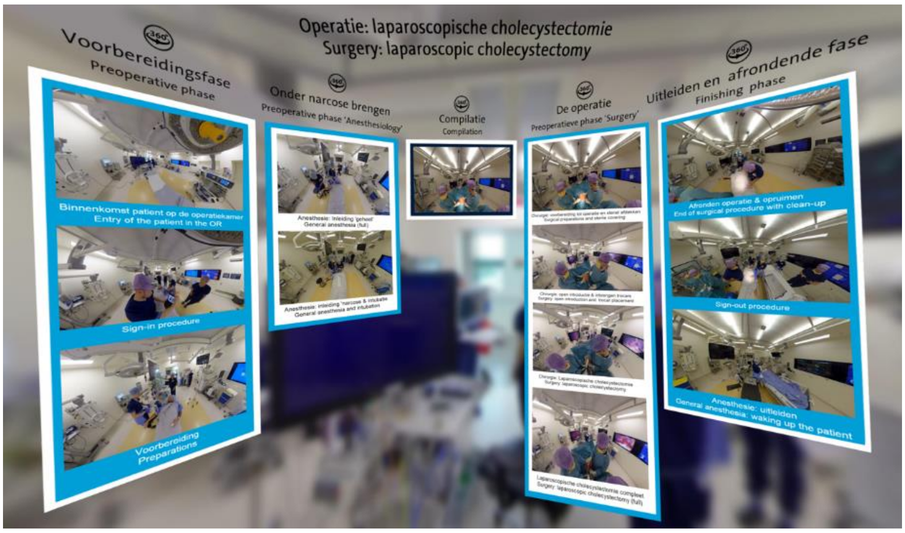

Appendix 3b. Project 2

Increasing Learning Return from the "Exchange of Cases and Practice Experiences" Sessions During the General Practice Internship

Context and educational problem
The participant chose to focus their UTQ project on medical students during their General Practice (GP) internship and the 'Exchange of Cases and Practice Experiences' sessions. These sessions were often passive, unstructured and yielded limited learning outcomes. The UTQ participant aimed to make these sessions more engaging and interactive and to improve clinical reasoning in common primary care situations.

Proposed innovation
Development of three interactive teaching methods:

1) "30 Seconds: Minor Ailments" game: Students describe diseases within 30 seconds using symptoms and features and their peers guess and give feedback
2) "Mr. A.I." virtual patient: Students question an AI-based virtual patient to reach diagnosis and treatment which is followed by peer, AI, and tutor feedback,
3) "Run your Opinion" debate game: Students take positions ("agree/disagree") on statements about GP or social medicine topics, explain arguments, and reflect together. These teaching methods were expected to increase student motivation by increasing students' autonomy, competence and relatedness according to the Self-Determination Theory and connecting to the PBL principles of constructive, collaborative, contextual and self-directed learning.

Implementation, evaluation and future vision
The pilot would be launched with one or two tutor groups and evaluated via student and tutor feedback and the end-of-rotation survey. Depending on the evaluations, the teaching methods could be adopted across all GP internship groups. Possible development of a digital platform for independent practice with virtual patients.

Patient Simulation BOT 40

PatientSimulationBOT

Conducts fictional patient interviews with a fictional GP.

Good morning!

Ask a question

PatientSimulationBOT 4o

To share

Good morning doctor!

What can I do for you?

I have been suffering from a burning sensation when urinating for a few days now.

b

Ask a question
